# Analysis of national physical activity and sedentary behaviour policies in China

**DOI:** 10.1186/s12889-023-15865-8

**Published:** 2023-05-30

**Authors:** Sitong Chen, Jintao Hong, Karen Milton, Bojana Klepac, Jiani Ma, Zeljko Pedisic

**Affiliations:** 1 Institute for Health and Sport, Victoria University, Melbourne, 3011 Australia; 2 Shanghai Research Institute of Sports Science (Shanghai Anti-Doping Agency), Shanghai, 200030 China; 3 Norwich Medical School, Faculty of Medicine and Health Sciences, University of East Anglia, Norwich, NR4 7TJ UK; 4 Mitchell Institute for Education and Health Policy, Victoria University, Melbourne, 3000 Australia; 5 Centre for Sport, Exercise and Life Sciences, Coventry University, Coventry, CV1 5FB UK; 6 School of Health and Social Development, Deakin University, Geelong, 3220 Australia; 7 Institute for Physical Activity and Nutrition, Deakin University, Geelong, 3220 Australia

**Keywords:** Health policy, Physical inactivity, Policy audit, Policy landscape, China

## Abstract

**Purpose:**

The aims of this study were to advance knowledge on physical activity (PA) and sedentary behaviour (SB) policies in China and to highlight related gaps and opportunities in the Chinese policy landscape.

**Methods:**

Literature and web-based searches were performed to identify national PA and SB policies in China. We assessed which of the 17 elements of the Health-Enhancing Physical Activity Policy Audit Tool (HEPA PAT, version 2) are included in each of the policy documents and whether and how they address the ‘cornerstones’ of PA and SB policy: PA and SB guidelines, targets, surveillance and monitoring, and public education programmes.

**Results:**

We found 60 national PA and SB policies, of which 54 focused on PA only and 6 focused on both PA and SB. There was a rapid increase in the number of policies issued between 2002 and 2021. In totality, the policies include all 17 key elements for a successful national policy approach to PA promotion according to the HEPA PAT. The policies reflect engagement from a range of sectors and encompass PA targets, recommendations for PA and SB, mandates and recommendations for school-related PA, plans for public education on PA, and plans for surveillance and monitoring of PA and SB.

**Conclusion:**

Our findings demonstrate that there has been increasing focus on PA and SB policies in China, which reflects efforts by policymakers to address the health burden of insufficient PA and excessive SB. More emphasis may be placed on SB in Chinese policy, particularly in terms of setting specific targets for population SB. Policymakers and other relevant public health stakeholders in China could also consider developing or adopting the 24-hour movement guidelines, in accordance with recent trends in several other countries. Collaboration and involvement of different sectors in the development and implementation of Chinese PA and SB policies should continue to be facilitated as part of a whole-of-system approach to health promotion.

**Supplementary Information:**

The online version contains supplementary material available at 10.1186/s12889-023-15865-8.

## Introduction

Insufficient physical activity (PA) is among the leading risk factors for global mortality and morbidity [1, 2]. Conversely, regular participation in PA plays an integral role in noncommunicable disease prevention. A large body of evidence demonstrates a range of health benefits of PA across the lifecourse, such as prevention of heart disease, stroke, type 2 diabetes, and different types of cancer in adults [3] and improved obesity status, cardiorespiratory fitness, and cognitive function in children and adolescents [4].

In addition to insufficient PA, another highly relevant but distinct lifestyle component—sedentary behaviour (SB)—has been recognised as a risk factor for ill-health [4, 5]. SB includes waking behaviours performed in lying, reclining, or sitting posture that require low energy expenditure of ≤ 1.5 metabolic equivalents [6]. SB is negatively associated with cardiometabolic health, mental health, and weight status [4, 5]. Owing to the benefits of PA and the adverse effects of SB, the World Health Organization (WHO) and many national health agencies have issued guidelines on recommended levels and/or patterns of PA and SB for different population groups [2, 7, 8]. However, a relatively high proportion of the global population do not meet the guidelines [9, 10]. This demonstrates an urgent need for scaled-up actions to address this global public health issue.

To address population levels of PA and SB, many international and collaborative strategic frameworks have been developed, including the *Global action plan on physical activity 2018–2030: more active people for a healthier world* (GAPPA) [11]. These types of strategic frameworks are important in addressing levels of PA and SB at the macro level [12–14]. However, to facilitate population behaviour change, they must be used to underpin policy at the national and subnational levels [15, 16]. The development and implementation of national-level PA and SB policy should ideally involve collaboration between relevant stakeholders across multiple sectors, including health, sport, transport, tourism, education, work and employment, environment, recreation and leisure, urban/rural design and planning, public finance, and research [17]. National policy can facilitate the creation of supportive environments for people to be physically active, as well as attractive and varied programmes and opportunities for PA [17, 18].

According to Klepac Pogrmilovic and colleagues [17], PA and SB policy refers to the “*totality of formal written policies, unwritten formal statements, written standards and guidelines, formal procedures, and informal policies (or lack thereof) that may directly or indirectly affect community- or population-level PA and SB*”. PA and SB policy analysis is defined as “*any kind of policy-relevant research that audits or assesses one or more aspects of PA and SB policy*”. An increasing number of analyses on PA and SB policy have been conducted to identify policy gaps and opportunities, inform policymakers, and facilitate changes to policies to improve their effectiveness [12, 19]. Owing to distinctive political structures and systems in different countries, there is merit in undertaking policy analysis at the national level. Such national-level policy analysis may help advance PA promotion in a given country.

Survey data demonstrate the need to promote more PA and less SB in the Chinese population [20–24], particularly because PA levels among Chinese adults appear to be decreasing over time [25]. Given that nearly 20% of the world’s population lives in China, significant contributions to global health can be expected from addressing the issue of insufficient PA in this country alone.

A range of studies have stressed the importance of effective PA policies to tackle the pressing issue of low PA in the Chinese population [26–29]. Accordingly, several PA policies have been developed in China, such as the *Healthy China 2030 Plan* [30] and *Healthy China Initiative (2019–2030)* [31]. These national-level policies include specific goals to promote PA and reduce SB at the population level. However, a structured scientific analysis of national PA and SB policies in China, the most populous country in the world, has not been conducted.

The aims of this study were to: (1) advance knowledge on PA and SB policies in China; and (2) highlight related gaps and opportunities in the Chinese policy landscape.

## Methods

### Study design

In this study, we focused on China’s national-level PA and SB policies, operationalised as any formal written policies, standards and guidelines (i.e. policy documents) aimed at increasing PA and reducing SB levels in the Chinese population. We undertook an audit of policies, focusing on their availability and content.

### Search strategy

Thorough literature and web-based searches were performed to identify national PA and SB policies in China. The web-based search focused on the authorized and official policies issued by the Communist Party of China and the State Council of the People’s Republic of China (i.e. the central administrative agency). These are the main institutions in China that have functions and powers to promote public health and issue national-level health policies related to PA and SB [32–35]. In our search we covered four policy sectors - health, education, culture, and sport - because Chinese policies that are primarily focused on the promotion of PA and SB are issued by governmental bodies in these sectors. Titles of all publicly available policies found on their official websites were screened. The initial search was conducted between the 1st and 15th of January, 2020. The literature search focused on the electronic literature database of the Chinese National Knowledge Infrastructure [36]. We searched for PA and SB policies using Chinese terms for “guideline” or “recommendation” combined with “exercise” or “physical activity” or “sedentary” or “screen”. At the end of 2021, updated searches were conducted to identify potentially eligible policies issued in 2021.

### Inclusion criteria and selection of relevant policies

**Fig. 1:**
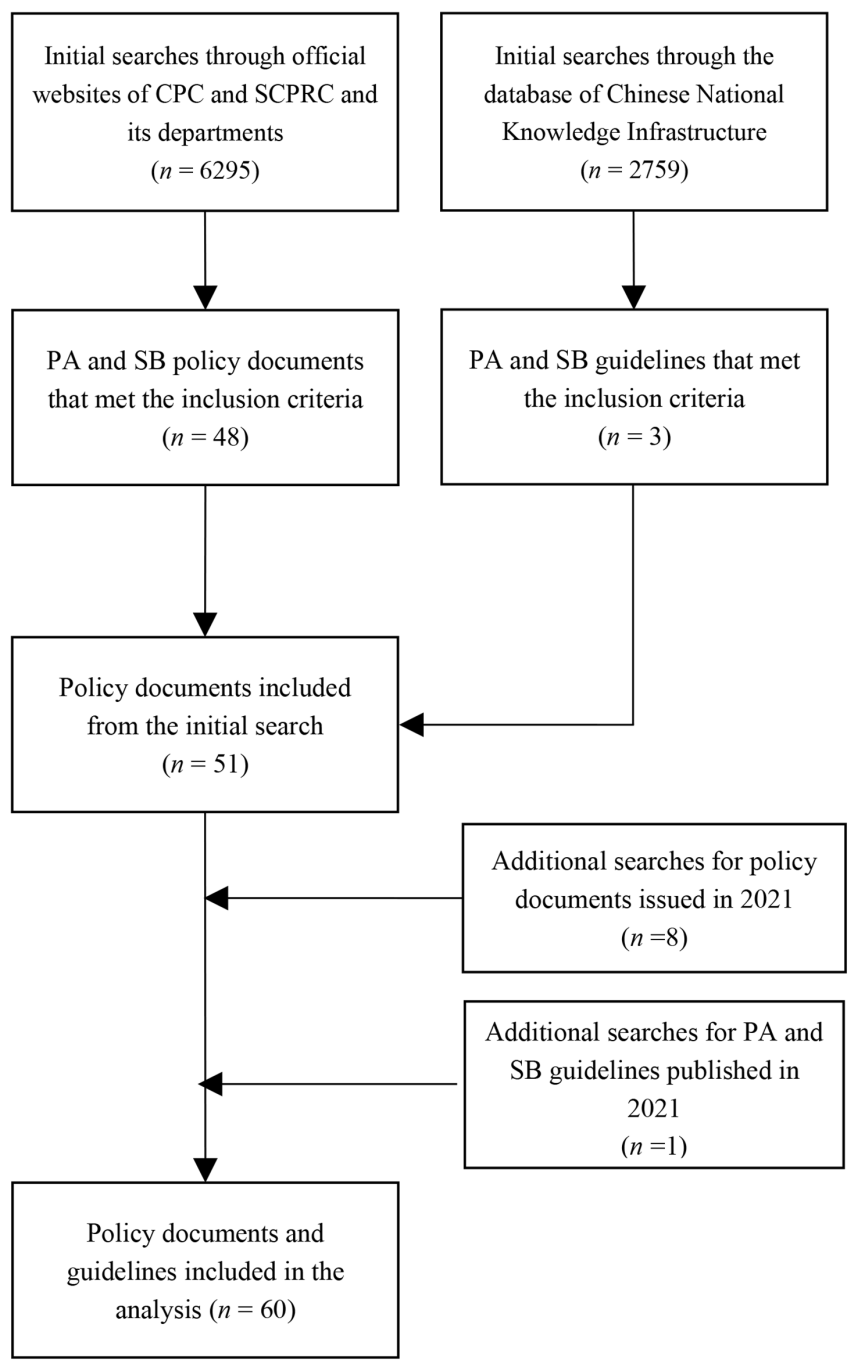
Flow chart of the policy search and selection process

We included policy documents that: (1) contain strategies, plans, or approaches to addressing PA or SB at the population level; (2) include one or more of the following terms (in Chinese) in their title or main body text: “health”, “education”, “fitness”, “exercise”, “physical activity”, “sport”, “physical education”, “sedentary” and “obesity”. We also included guidelines on PA and SB issued by leading Chinese health organizations (i.e. Centre for Disease Control) or research institutes (i.e. medical or sport university). To be considered for inclusion, a document had to have national scope. We included both past and present policies. Two authors participated in the process of policy identification and selection (SC and JH). They independently assessed each policy against the eligibility criteria, and any disagreements between their assessments were resolved through discussion. The search and selection processes are depicted in Fig. 1.

### Data extraction and analysis

We first extracted information on title, publication year, issuing body, and web link of each policy document. We then used the second version of the Health-enhancing Physical Activity Policy Audit Tool (HEPA PAT) to assess the policies. HEPA PAT identifies 17 key elements for a successful national policy approach to PA promotion [37]. We first determined which of the elements are included in each of the policy documents. We then analysed the following four domains, which are recognised as ‘cornerstones’ of PA and SB policy: national guidelines on PA and SB; national PA and SB goals and targets; national PA and SB surveillance and monitoring; and public education programmes on PA and SB [38, 39]. As part of this analysis, we recorded: (1) whether the policy refers to any of the four domains; (2) which target group(s) the policy refers to; (3) which settings and activity types the included PA and SB recommendations refer to; (4) whether the PA and SB recommendations refer to frequency, duration, and intensity of activity; (5) what the content of the educational programmes on PA and SB included; and (6) what the planned mode of delivery for the educational programmes was. Data extraction and analysis were performed independently by two authors (SC and JH), and any disagreements were resolved through discussion.

## Results

### Search and policy selection results

By screening a total of 6,295 policy documents on the Communist Party of China and the State Council of the People’s Republic of China websites and its four affiliated sectors, we identified 48 written policies and guideline documents that met the inclusion criteria (Fig. 1). Through screening 2759 documents in the database of the Chinese National Knowledge Infrastructure, three additional PA and SB guidelines were identified as eligible for inclusion. In the search update, we found an additional nine PA and SB policies, resulting in a total of 60 policy documents eligible for inclusion.

### General characteristics of the included policies

**Fig. 2:**
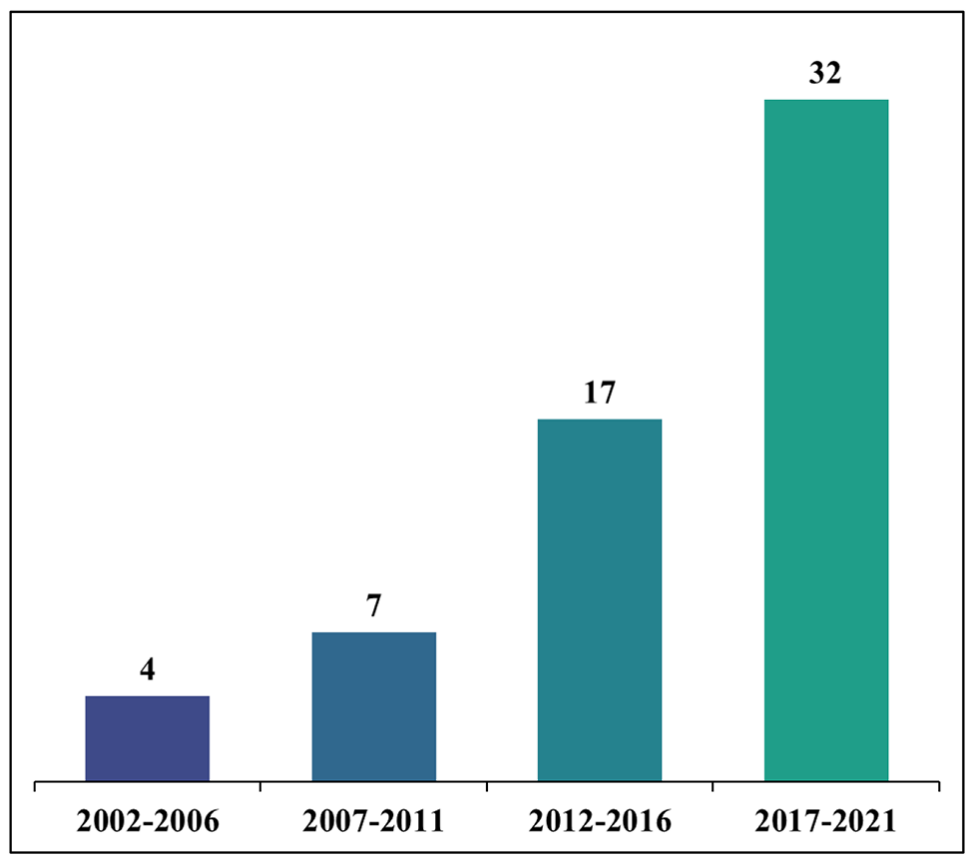
Number of Chinese physical activity and sedentary behaviour policies by the time of their publication

**Fig. 3:**
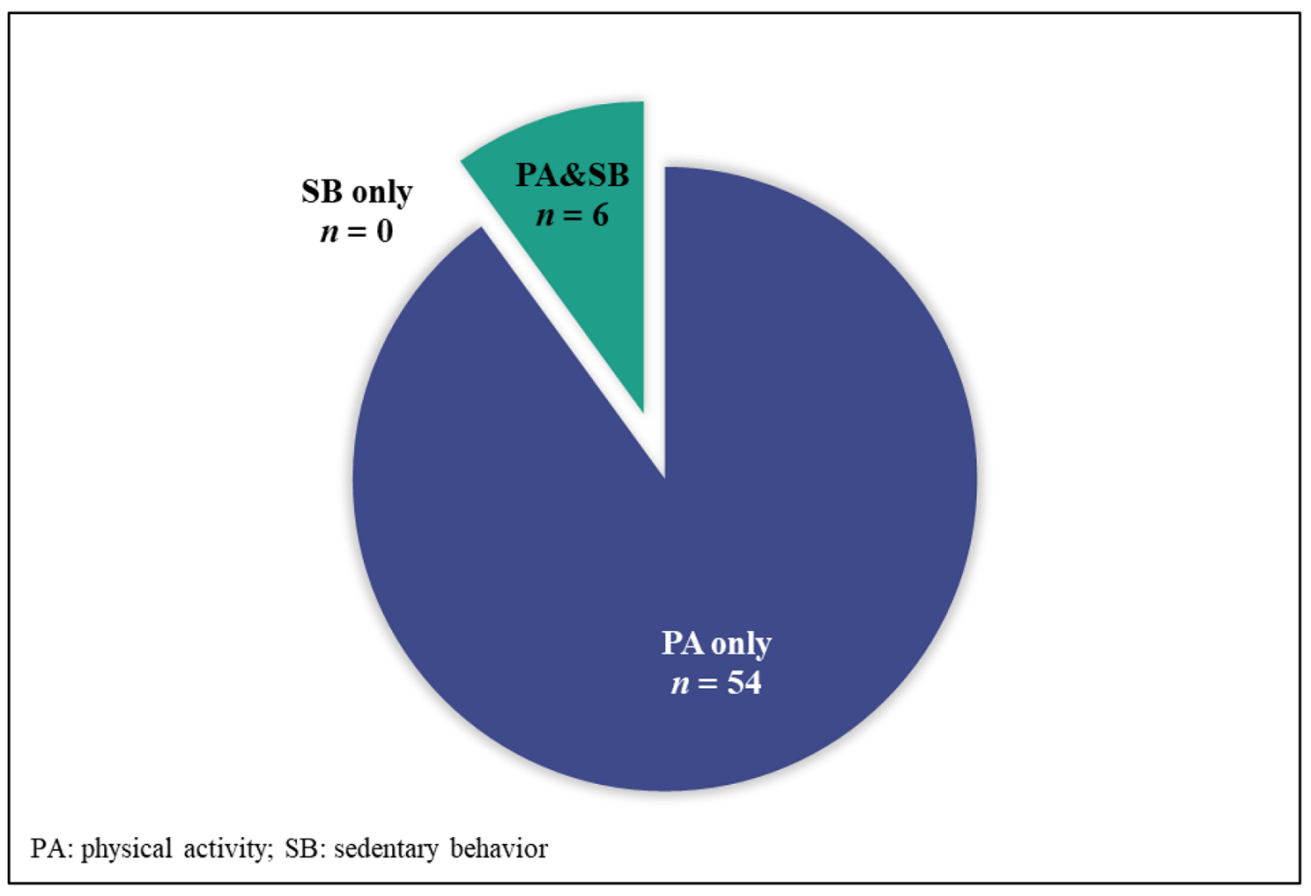
The focus of Chinese physical activity and sedentary behaviour policies

Of all the included policies (*n* = 60), the earliest was issued in 2002 and the latest was issued in 2021 (Additional file 1). Additional file 2 shows that different sectors have issued the policies. The General Administration of Sport of China, the State Council of the People’s Republic of China, and the Ministry of Education were involved in the development of 25, 23 and 13 policies, respectively (with some policies co-produced by multiple agencies). Figure 2 shows a rapid increase in the number of policies issued between 2002 and 2021. Fifty-four policies focused on PA only, six policies focused on both PA and SB, but none of the policies focused on SB only (Fig. 3).

### Key elements for a successful national policy approach to PA promotion

Individual policies included between three and 15 (median = 10) key elements for a successful national policy approach to PA promotion from the HEPA PAT (Additional file 3). The following nine elements were present in most policies: consultative approach in development *(n* = 60); integration across other sectors and policies (*n* = 59); multiple strategies (*n* = 51); political commitment (*n* = 55); ongoing funding (*n* = 39); leadership and coordination (*n* = 55); working in partnership (*n* = 58); links between policy and practice (*n* = 56); and network supporting professionals (*n* = 51). An implementation plan with a specified time frame for implementation was included in 50% of the policies. Other elements were also present, albeit they were represented in fewer policies.

### Four cornerstones of PA and SB policy

In terms of the four cornerstones of PA and SB policy, five policies included PA or SB recommendations, 20 policies specified national goals and targets for PA, 17 mentioned surveillance and monitoring, and 15 involved public education (operationalised as “communication strategy” in the HEPA PAT).

#### PA and SB recommendations

Recommendations for PA and SB were provided in five policy documents, out of which four were PA and SB guidelines documents (Table 1). The recommendations targeted preschool children, school-age children and adolescents, adults, older adults, people with chronic conditions, and pregnant women. The recommendations covered overall PA, aerobic activity, structured/organised PA, daily steps, muscle-strengthening exercise, bone-strengthening activity, stretching, outdoor play, overall SB, and screen time, specifying their preferable frequency, duration and intensity, where applicable. Two policies referred specifically to outdoor activities (policy 38 and 60; Additional file 1).

**Table 1:** Physical activity and sedentary behaviour recommendations in Chinese policies

Characteristic	Category	n*	%	Policy^†^
**Includes recommendations**	Yes	5	8.3	10, 17, 38, 39, 60
	No	55	91.7	1, 2, 3, 4, 5, 6, 7, 8, 9, 11, 12, 13, 14, 15, 16, 18, 19, 20, 21, 22, 23, 24, 25, 26, 27, 28, 29, 30, 31, 32, 33, 34, 35, 36, 37, 40, 41, 42, 43, 44, 45, 46, 47, 48, 49, 50, 51, 52, 53, 54, 55, 56, 57, 58, 59
**Type of document**	Guidelines	4	6.7	10, 38, 39, 60
	Other	1	1.7	17
**Target group**	Preschool children	2	3.3	38, 60
	School-age children and adolescents	2	3.3	39, 60
	Adults	2	3.3	10, 60
	Older adults	3	5.0	10, 17, 60
	People with chronic conditions	2	3.3	10, 60
	Pregnant women	1	1.7	60
**Setting**	Non-specific	4	6.7	10, 38, 39, 60
	Outdoors	2	3.3	38, 60
**Activity type**	Overall physical activity	4	6.7	10, 38, 39, 60
	Aerobic activity	4	6.7	10, 38, 39, 60
	Structured/organised activity^‡^	1	1.7	10
	Daily steps	1	1.7	10
	Muscle-strengthening exercise	3	5.0	10, 39, 60
	Bone-strengthening activity	3	5.0	38, 39, 60
	Stretching	2	3.3	10, 60
	Outdoor play	2	3.3	38, 60
	Overall sedentary behaviour	3	5.0	38, 39, 60
	Screen time	3	5.0	38, 39, 60
**Dose**	Frequency	4	6.7	10, 38, 39, 60
	Duration	4	6.7	10, 38, 39, 60
	Intensity	4	6.7	10, 38, 39, 60

* Number of policies

^†^ The names of policies are provided in Additional file 1

^‡^ Structured/organised activity refers to morning exercise or exercise at workplace

In addition to these general PA recommendations, school-related PA was mentioned in approximately 30% of policies (Table 2). This included policies/recommendations on PA as part of physical education classes (*n* = 17), school sport participation (*n* = 17), and PA during recess (*n* = 16). While all the policies referred to the frequency and duration of physical education classes, only two of them referred specifically to the intensity of PA.

**Table 2:** School-related physical activity and sedentary behaviour policies/recommendations

Characteristic	Category	n*	%	Policy^†^
**Includes school-related policies/recommendations**	Yes	17	28.3	3, 4, 5, 7, 11, 13, 18, 21, 23, 26, 30, 32, 34, 44, 45, 49, 56
	No	43	71.7	1, 2, 6, 8, 9, 10, 12, 14, 15, 16, 17, 19, 20, 22, 24, 25, 27, 28, 29, 31, 33, 35, 36, 37, 38, 39, 40, 41, 42, 43, 46, 47, 48, 50, 51, 52, 53, 54, 55, 57, 58, 59, 60
**Activity type**	Physical activity as part of physical education classes	17	28.3	3, 4, 5, 7, 11, 13, 18, 21, 23, 26, 30, 32. 34, 44, 45, 49, 56
	School sport participation	17	28.3	3, 4, 5, 7, 11, 13, 18, 21, 23, 26, 30, 32, 34, 44, 45, 49, 56
	Physical activity during recess time at school	16	26.7	4, 5, 7, 11, 13, 18, 21, 23, 26, 30, 32, 34, 44, 45, 49, 56
**Dose**	Frequency	17	28.3	3, 4, 5, 7, 11, 13, 18, 21, 23, 26, 30, 32, 34, 44, 45, 49, 56
	Duration	17	28.3	3, 4, 5, 7, 11, 13, 18, 21, 23, 26, 30, 32, 34, 44, 45, 49, 56
	Intensity	2	3.3	26, 45

* Number of policies

^†^ The names of policies are provided in Additional file 1

#### National targets for PA and SB

One third of included policies (*n* = 20) set specific targets for PA promotion (Table 3). Most of these policies set targets for children and adolescents (*n* = 12), and nearly half (*n* = 9) set targets for the general population.

**Table 3:** Physical activity and sedentary behaviour targets specified in Chinese policies

Characteristic	Category	n*	*%*	Policy^†^
Includes target(s)	Yes	20	33.3	4, 5, 7, 11, 18, 22, 23, 24, 25, 26, 30, 33, 34, 43, 44, 45, 49, 54, 56, 58
	No	40	66.7	1, 2, 3, 6, 8, 9, 10, 12, 13, 14, 15, 16, 17, 19, 20, 21, 27, 28, 29, 31, 32, 35, 36, 37, 38, 39, 40, 41, 42, 46, 47, 48, 50, 51, 52, 53, 55, 57, 59, 60
Target behaviour	Physical activity	20	33.3	4, 5, 7, 11, 18, 22, 23, 24, 25, 26, 30, 33, 34, 43, 44, 45, 49, 54, 56, 58
	Sedentary behaviour	0	0.0	/
Target group	School-age children and adolescents	12	20.0	4, 5, 7, 11, 18, 23, 26, 30, 34, 49, 54, 56
	General population	9	15.0	22, 24, 25, 30, 33, 43, 44, 45, 58

* Number of policies

^†^ The names of policies are provided in Additional file 1

#### Surveillance and monitoring of PA and SB

Almost 30% of the policies mentioned surveillance or monitoring of PA and/or SB (Table 4). Twenty-two per cent of policies referred to the general population, while only 5% referred to school-age children and adolescents and 1.7% to preschool children. PA was mentioned as an outcome of assessment more commonly than SB (in 17 vs. in 14 policies).

**Table 4:** Surveillance and monitoring of physical activity and sedentary behaviour planned within Chinese policies

Characteristic	Category	n*	%	Policy^†^
**Mentions surveillance or monitoring**	Yes	17	28.3	1, 21, 22, 23, 24, 26, 28, 30, 38, 45, 46, 47, 51, 54, 56, 57, 58
	No	43	71.7	2, 3, 4, 5, 6, 7, 8, 9, 10, 11, 12, 13, 14, 15, 16, 17, 18, 19, 20, 25, 27, 29, 31, 32, 33, 34, 35, 36, 37, 39, 40, 41, 42, 43, 44, 48, 49, 50, 52, 53, 55, 59, 60
**Target group**	Preschool children	1	1.7	38
	School-age children and adolescents	3	5.0	23, 54, 56
	General population	13	21.7	1, 21, 22, 24, 26, 28, 30, 45, 46, 47, 51, 57, 58
**Indicator**	Physical activity	17	28.3	1, 21, 22, 23^‡^, 24, 26, 28, 30, 38, 45, 46, 47, 51, 54^‡^, 56^‡^, 57, 58
	Sedentary behaviour	14	23.3	1, 21, 22, 24, 26, 28, 30, 38, 45, 46, 47, 51, 57, 58

* Number of policies

^†^ The names of policies are provided in Additional file 1

^‡^ Physical education, sport and exercise in the school setting

#### Public education for PA and SB

Public education for PA was mentioned in 25% of the policies (Table 5). With regard to the target population, the most represented public education policies were for school-age children and adolescents (*n* = 6), followed by the general population (*n* = 3), older adults (*n* = 2), and people with chronic conditions (*n* = 2). Public education for PA for other population groups, including preschool children, adults, employees, and pregnant women were mentioned in one policy each. Five public education policies contained information on both “how much” and “what type” of activity should be undertaken, while three of them also contained information on “why” it is important to be physically active and/or reduce SB. Media or mode of message delivery for public education on PA and/or SB was specified in 15% of policies, while provider or source of message delivery was mentioned in 5% of policies.

**Table 5:** Public education on physical activity planned within Chinese policies

Characteristic	Category	n*	%	Policy^†^
**Mentions public education**	Yes	15	25.0	3, 4, 5, 8, 10, 13, 14, 17, 20, 35, 38, 39, 41, 46, 60
	No	45	75.0	1, 2, 6, 7, 9, 11, 12, 15, 16, 18, 19, 21, 22, 23, 24, 25, 26, 27, 28, 29, 30, 31, 32, 33, 34, 36, 37, 40, 42, 43, 44, 45, 47, 48, 49, 50, 51, 52, 53, 54, 55, 56, 57, 58, 59
**Target population**	Preschool children	1	1.7	38
	School-age children and adolescents	6	10.0	3, 4, 5, 13, 39, 41
	Adults	1	1.7	10
	Older adults	2	3.4	14, 17, 20
	General population	3	5.0	35, 46, 60
	Employees	1	1.7	8
	Pregnant women	1	1.7	60
	People with chronic conditions	2	3.4	10, 60
**Type of information included**	How much	5	8.3	10, 17, 38, 39, 60
	What type	5	8.3	10, 17, 38, 39, 60
	Why	3	5.0	10, 38, 60
**Media or mode of message delivery**	Specified	9	15.0	8, 10, 13, 14, 20, 35, 38, 41, 46
	Not specified	6	10.0	3, 4, 5, 17, 39, 60
**Provider or source of message delivery**	Specified	3	5.0	3, 8, 10
	Not specified	12	20.0	4, 5, 13, 14, 17, 20, 35, 38, 39, 41, 46, 60

* Number of policies

^†^ The names of policies are provided in Additional file 1

## Discussion

### Key findings

The key finding of this study is that the number of PA and SB policies in China has substantially increased over the past two decades, currently counting as many as 60 policies. Considerably fewer policies focused on SB than on PA, which is consistent with previous findings [12, 40]. The policies reflect engagement from a range of sectors. They encompass PA targets, recommendations for PA and SB, mandates and recommendations for school-related PA, plans for public education on PA, and plans for surveillance and monitoring of PA and SB. In totality, the policies include all key elements for a successful national policy approach to PA promotion according to the HEPA PAT.

### Key stakeholders in the policy development

The present study demonstrates that the importance of PA and SB policy has been recognised by China’s policymakers. In the face of insufficient PA and excessive SB in the Chinese population [27, 41], China’s policymakers have made increasing efforts to address this public health issue, which is consistent with international trends [12, 40] and reflects a solid political traction. After winning the bid to host the 2008 Olympic Games in Beijing, the Chinese government decided to use this opportunity to further develop national PA policies and increase investments in PA promotion [32, 33]. This led to an increase in population levels of PA in the subsequent 14 years [42]. This success further promotes the case for policymakers in China and other countries to develop national policies and increase investments in PA [43].

The central administrative authorities (Communist Party of China and State Council of the People’s Republic of China) and the governmental bodies in the sport and education sectors were the leaders in PA and SB policy development, while the ministries of transportation, urban design and environment were less represented, which is consistent with findings from other countries [40]. This suggests a need to engage policymakers from more key sectors, given that population PA and SB are influenced by a broad range of intrapersonal, social, environmental, and political factors [44]. Thus, collaborative involvement of different sectors, organisations, and agencies should continue to be facilitated as part of a whole-of-system approach to PA promotion [17, 45]. Such an approach is emphasised in the *Healthy China 2030 Action Plan*—a landmark policy that integrates multidimensional and multisectoral resources to tackle physical inactivity [26–28, 46]. However, our study did not explore the extent to which a coordinated whole-of-system approach is being taken to implement the policy. Therefore, further research into characteristics of policy implementation in China is needed, as such research may inform policy improvements.

### Key elements for a successful national policy approach to PA promotion

When assessing PA and SB policies against the 17 key elements for a successful national policy approach to PA promotion outlined in the HEPA PAT [37], not every individual policy has to include all the elements. Instead, according to the HEPA PAT, it is recommended that all 17 elements are represented in the overall national PA policy (i.e. in the totality of all individual policies). Overall, the Chinese PA and SB policies include all the elements, similar to policies in Poland [47], Germany [48], the Netherlands [48] and Ireland [48]. The Chinese policy landscape can therefore be considered as supportive for the promotion of PA. An element that was covered, but that received less attention than others, was evaluation (including evaluation of policies, interventions, and economic burden of physical inactivity); hence this is an area that might be strengthened in future policies.

### Four cornerstones of PA and SB policy

#### PA and SB recommendations

Since 2011, four national guidelines on PA and SB have been issued in China, the latest of which was published in 2021. When examining the content of the guidelines, they are consistent with other guidelines such as those from the WHO [2], and the US [49]. The guidelines provide Chinese people with evidence-based guidance on PA participation and information on the health impacts of SB. In addition, the latest national PA guidelines that were published in 2021, included for the first time specific recommendations for pregnant women [50], which demonstrates an increased awareness among policymakers and other public health stakeholders of the importance of promoting PA among different population groups. However, recommendations for some population groups (e.g., people with HIV and hypertension) that are part of the latest WHO guidelines [2] are yet to be included in the Chinese policies. However, in isolation, the PA and SB guidelines are unlikely to change population behaviour. Translating the guidelines into practice is the next key step to maximising their potential health impact [51]. In addition, there is an international trend towards integrated movement-behaviour guidelines including recommendation for PA, SB and sleep in combination [52–60]. Future Chinese policies may consider developing or adopting 24-hour movement guidelines that include integrated recommendations on PA, SB, and sleep [50].

In addition to the general PA and SB recommendations, some policies recommend or stipulate the frequency, duration and intensity of specific types of PA in the school setting. This is because China’s education sector has a strong commitment to increasing PA through the school setting. For example, some policies require “school-age children and adolescents to have at least 60 minutes of sports, physical education, and exercise activities daily on schooldays” (i.e. policies 11, 13 and 18, Additional file 1). Compared with similar policies in many European countries, this is a relatively high requirement [61]. Only two Chinese school-related PA and SB policies, mentioned intensity of PA. Only referring to the frequency and duration of school-related PA presents a challenge for promoting physical fitness and health, because PA intensity also plays a key role [4]. The inclusion of recommendations on intensity in future school-related PA policies should be encouraged where possible. It is expected that such policies will support children and adolescents to achieve recommended levels of PA. However, there may be a compensatory effect, whereby, due to more PA in school, children and adolescents reduce their leisure-time PA outside of school hours [62, 63]. It is important to acknowledge that the compensation effect would not be an issue if the added level of school PA was sufficient for children to meet the PA guidelines. Furthermore, “International School-Related Sedentary Behaviour Recommendations” have recently been developed [64] and translated to Chinese. Policymakers in China may wish to consider developing recommendations on SB in the school setting or adopting the above-mentioned recommendations.

#### National targets for PA and SB

Analysing the national targets set in policies is important to understand what policymakers expect to achieve with the promotion of more PA and less SB and which population groups they think should be prioritised. Specific targets set for school-age children and adolescents as a priority population group in a dozen Chinese policies (*n* = 12) is an important finding of our study, reflecting policymakers’ expectations on the health behaviours and development in this age group. Accordingly, schools were the most frequently mentioned setting, particularly in the policies developed and issued by the Ministry of Education. The goals of China’s school-related PA and SB policies are to increase participation of children and adolescents in structured/organized activity in the school setting, which may affect overall levels of PA in this age group. Comparatively, somewhat lower number of policies (*n* = 9) specified targets for the general population, including adults. Also, no policy included targets for SB, which could be considered in the development of future policies.

#### Surveillance and monitoring of PA and SB

Surveillance and monitoring of PA and SB can inform and guide national actions to promote active lifestyles [65]. We found that SB surveillance was mentioned less frequently in national policies, compared with PA surveillance, which is consistent with findings from other countries [40]. This can be explained by the fact that PA has been a longstanding and widely acknowledged element of preventive health for several decades, while SB was recognised as a health risk factor more recently. While the “National Fitness Surveillance and Monitoring Project” and “Chinese Health and Nutrition Survey” assess both PA and SB levels, future policies in China may consider putting more emphasis on SB, particularly because PA and SB together with sleep represent co-dependent components of the daily time-use composition [66]. It is also recommended to establish specialised surveillance systems that are focused exclusively on these behaviours and associated intrapersonal, interpersonal, environmental, societal, and policy factors [67].

#### Public education for PA and SB

Public education has been recognised as important in enhancing the health impacts of PA and SB policy [13, 14, 68, 69]. Through public education it is possible to disseminate transparent and reliable messages about the health impacts of PA and SB [70, 71], aimed at increasing knowledge and awareness and motivating behaviour change in the population. We found that in Chinese policies, school-age children and adolescents were the most targeted population group in terms of public education, reflecting the focus of Chinese policy on this group. While more than a dozen Chinese policies mention public education for PA and/or SB, not all of them include information on “how much”, “what type”, and “why”. Some of the policies also do not specify the provider and mode of message delivery. According to the PA messaging framework and checklist [72], to maximise the impact of public education on PA and SB, future policies should consider including specific statements on the above-mentioned components of message delivery. Given that policies emphasised the importance of knowledge and understanding of PA and SB as health factors, it would also be important to explore how many Chinese people understand the health effects of these behaviours or know the associated recommendations.

### Strengths and limitations

The current study has two key strengths: (1) we conducted a systematic search of Chinese national-level policies; and (2) we undertook an in-depth analysis of a large number of relevant national policies using existing analytical approaches. However, some study limitations should be acknowledged.

The study has several limitations. First, the study was limited to policies that were accessible online, that is, through the Internet searches. Therefore, it is possible that some PA and SB policies that would meet our eligibility criteria were missed. Second, we focused exclusively on formal written policies, standards and guidelines (i.e. policy documents). For a more comprehensive analysis of Chinese PA and SB policy, future studies should also consider unwritten formal statements, formal procedures, and informal policies [17]. Third, we examined only national-level PA and SB policies. Due to a very large number of provinces, autonomous regions and municipalities (*n* = 32), cities (*n* = 662), and counties (*n* = 1355) in mainland China, it was beyond the scope of this study to analyse provincial and local policies. Fourth, in our search, we focused on health, education, culture, and sport sectors, and used only the search terms that are likely to be found in PA and SB policies issued within these sectors. Policies that directly or indirectly influence PA and SB in the population may also be issued in a range of other sectors, including environment, public finance, research, tourism, transport, urban/rural planning and design, and work and employment [17]. In our search we did not use some common terms related to PA such as “walking”, “cycling”, and “active travel”, often mentioned in transport policies, nor terms such as “obesity”, “heart disease”, and “diabetes”, associated with noncommunicable disease policies. Therefore, in this analysis, we may have missed some relevant PA and SB policies. Fifth, we focused our analysis on the 17 elements of the HEPA PAT and the four ‘cornerstones’ of PA policy. These tools predominantly focus on PA as opposed to SB and therefore some information related to SB may have been missed. In addition, utilising an alternative framework, such as the Comprehensive Analysis of Policy on Physical Activity (CAPPA) framework may have elicited additional or different information [17]. Also, the development of HEPA PAT was informed by findings of pilot tests in European countries only. Nevertheless, given the broad scope of its items, the tool has been successfully applied in various countries outside Europe [73, 74]. Sixth, we solely focused on the availability and content of PA and SB policies in China. Future studies should analyse the implementation and effectiveness of Chinese PA and SB policies. Finally, the current study used general, non-country specific analytical approaches to assess PA and SB policies. Some specific features of Chinese policies may have therefore been missed. However, the use of the standardised analytical approaches enabled us to compare Chinese data with findings from other countries. Future studies should consider adopting assessments or analytical approaches to examine PA and SB policies that are tailored to the Chinese context.

## Conclusion

Our findings demonstrate that there has been increasing focus on PA and SB policies in China, which reflects efforts by policymakers to address the health burden of insufficient PA and excessive SB. The overall Chinese PA and SB policy landscape includes all key elements for a successful national policy approach to PA promotion. It encompasses PA targets, recommendations for PA and SB, mandates and recommendations for school-related PA, plans for public education on PA, and plans for surveillance and monitoring of PA and SB. More emphasis may be placed on SB in Chinese policy, particularly in terms of setting specific targets for population SB. Policymakers and other relevant public health stakeholders in China could also consider developing or adopting the 24-hour movement guidelines, in accordance with recent trends in several other countries. Collaboration and involvement of different sectors in the development and implementation of Chinese PA and SB policies should continue to be facilitated as part of a whole-of-system approach to health promotion.

## Electronic supplementary material

Below is the link to the electronic supplementary material.

Additional file 1: Chinese physical activity and sedentary behavior policies included in the study

Additional file 2: Number of Chinese physical activity and sedentary behaviour policies per issuing body/institution

Additional file 3: Key elements for a successful national policy approach to physical activity promotion included in Chinese policies

## Data Availability

The datasets used and/or analyzed during the current study are available from the corresponding author on reasonable request.
