# Supplementary material for: Analysis of national physical activity and sedentary behaviour policies in China
Source: BMC Public Health. 2023 May 30;23:1024. doi: 10.1186/s12889-023-15865-8 (PMC10230767; doi:10.1186/s12889-023-15865-8)
Supplement: Supplementary file 1 — Additional file 1: Chinese physical activity and sedentary behavior policies included in the study [file 12889_2023_15865_MOESM1_ESM.docx]

**Additional file 1. Chinese physical activity and sedentary behavior policies included in the study**

| **No.** | **Title** | **Year** | **Issuing body** | **Web link** |
| --- | --- | --- | --- | --- |
| 1 | Opinions on Further Strengthening and Improving the Work Concerning Physical Culture and Sports in the New Era | 2002 | CCCPC, SCPRC | http://www.sport.org.cn/search/system/xgwj/2018/1108/191968.html |
| 2 | Interim Provisions on the Work Concerning Physical Culture and Sports in Rural Areas | 2002 | GASC, MARA | http://www.sport.org.cn/search/system/gfxwj/qzty/2018/1108/191847.html |
| 3 | Opinions on Further Strengthening the Work Concerning School Physical Education and Improving Students’ Physical Health | 2006 | MOE, GASC | http://old.moe.gov.cn/publicfiles/business/htmlfiles/moe/moe_942/200612/19106.html |
| 4 | The Decision of Implementing the Sunshine Sports Program for Hundreds of Millions of Students Nationwide | 2006 | MOE, GASC, CYLC | http://www.moe.gov.cn/s78/A17/twys_left/moe_938/moe_939/s3276/201001/t20100128_80877.html |
| 5 | Opinions on Strengthening Physical Education, Sports and Exercises for Enhancing Physical Health and Fitness of Children and Youth | 2007 | CCCPC, SCPRC | http://www.gov.cn/jrzg/2007-05/24/content_625090.htm |
| 6 | Opinions on Further Strengthening the Work Concerning Physical Culture and Sports for Persons with Disabilities | 2008 | GOSC | http://www.sport.org.cn/search/system/xgwj/2018/1108/191963.html |
| 7 | Outline of National Medium- and Long-Term Education Reform and Development Plan (2010–2020) | 2010 | MOE | http://old.moe.gov.cn/publicfiles/business/htmlfiles/moe/info_list/201407/xxgk_171904.html?authkey=gwbux |
| 8 | Opinions on Further Strengthening the Work Concerning Physical Culture and Sports for Staff and Workers | 2010 | GASC, ACFTU | http://www.sport.org.cn/search/system/gfxwj/qzty/2018/1108/191861.html |
| 9 | Opinions on Fulfilling the Functional Role of Comprehensive Cultural Stations in Towns and Rural Areas to Further Strengthen the Work Concerning Physical Culture and Sports in Rural Areas | 2010 | GASC, MOC, MARA | http://www.sport.org.cn/search/system/gfxwj/qzty/2018/1108/191859.html |
| 10 | Chinese Adults Physical Activity Guideline (Trial) | 2011 | NHC | http://www.gxcdc.com/uploadfile/2016/0601/20160601053607137.pdf |
| 11 | Provisions of Ensuring Daily 1-hour for Physical Education, Sports and Exercise in School of Students | 2011 | MOE | http://www.moe.gov.cn/srcsite/A17/s7059/201107/t20110708_171747.html |
| 12 | Opinions on Further Strengthening Cultural Development for the Elderly | 2012 | CNCA | https://www.mct.gov.cn/whzx/bnsj/ggwhs/201411/t20141106_764493.htm |
| 13 | Notice of Several Opinions on Further Strengthening the Work Concerning School Physical Education | 2012 | MOE, NDRF, MOF, GASC | http://www.gov.cn/zhengce/content/2012-10/29/content_5309.htm |
| 14 | Several Opinions on Promoting the Development of Health Services Industry | 2013 | SCPRC | http://www.sport.org.cn/search/system/xgwj/2018/1108/191952.html |
| 15 | Decision on Some Major Issues Concerning Comprehensively Deepening the Reform | 2013 | CCCPC | http://www.gov.cn/jrzg/2013-11/15/content_2528179.htm |
| 16 | Opinions on Accelerating and Advancing the Development of Health and Elderly Care Services | 2014 | NDRF, MCA, MOF, MLR, MOHURD, NHC, PBOC, SAT, GASC, CBRC | http://www.nhc.gov.cn/lljks/zcwj2/201806/f2d7ddd2f54846d1bb54f53c0f9d6784.shtml |
| 17 | Notice of Core Information about Elderly Health | 2014 | NHC | http://www.chinafpa.org.cn/zcfg/xgfg/201901/t20190125_42928.html |
| 18 | The Development Plan for Children in Impoverished Areas (2014-2020) | 2014 | GOSC | http://www.gov.cn/zhengce/content/2015-01/15/content_9398.htm |
| 19 | Opinions on Strengthening and Improving the Work Concerning Sports for All | 2014 | GASC | http://www.sport.gov.cn/n16/n1077/n1227/6062324.html |
| 20 | Opinions on Further Strengthening the Work Concerning Physical Culture and Sports for the Elderly under New Circumstances | 2015 | GASC, NDRF, MCA, MOF, MARA, MOC, NHC, NTA, CNCA, ACFTU, ACWF, CDPF | http://www.sport.org.cn/search/system/gfxwj/qzty/2018/1108/191874.html |
| 21 | Regulations on National Fitness (2016 Version) | 2016 | SCPRC | http://search.chinalaw.gov.cn/law/searchTitleDetail?LawID=334355&Query=%E5%85%A8%E6%B0%91%E5%81%A5%E8%BA%AB%E6%9D%A1%E4%BE%8B&IsExact= |
| 22 | The 13th Five-Year Plan for the Development of Physical Culture and Sports | 2016 | GASC | http://www.sport.org.cn/search/system/gfxwj/other/2018/1108/191840.html |
| 23 | Opinions on Strengthening School Physical Education to Promote Comprehensive Development of Students’ Physical and Mental Health | 2016 | GOSC | http://www.gov.cn/zhengce/content/2016-05/06/content_5070778.htm |
| 24 | National Fitness Program (2016-2020) | 2016 | SCPRC | http://www.gov.cn/zhengce/content/2016-06/23/content_5084564.htm |
| 25 | Winter Sports Development Plan (2016-2025) | 2016 | NDRF, GASC, MOE, NTA | http://www.gov.cn/xinwen/2016-11/25/content_5137611.htm |
| 26 | Outline of Healthy China 2030 Plan | 2016 | CCCPC, SCPRC | http://www.gov.cn/xinwen/2016-10/25/content_5124174.htm |
| 27 | Plan on Popularising and Promoting Mass Winter Sports (2016-2020) | 2016 | GASC, NDRF, MOE, MIIT, MCA, MOF, MOHRSS, MLR, MOHURD, MWR, MARA, MOC, PBOC, GAC, SAT, SAIC, NFGA, NTA, CBIRC, ACFTU, CYLC, ACWF, CDPF | http://www.gov.cn/xinwen/2016-11/07/content_5128878.htm |
| 28 | Law of the People's Republic of China on Physical Culture and Sports (2016 Amendment) | 2016 | NPCSC | http://search.chinalaw.gov.cn/law/detail?LawID=394870 |
| 29 | The 13th Five-Year Plan for Education | 2017 | SCPRC | http://www.gov.cn/zhengce/content/2017-01/19/content_5161341.htm |
| 30 | The 13th Five-Year Plan for Hygiene and Health | 2017 | SCPRC | http://www.sport.org.cn/search/system/xgwj/2018/1205/194911.html |
| 31 | The 13th Five-Year Plan for National Development Plan for the Elderly and Establishing Elderly Care Service System | 2017 | SCPRC | http://www.sport.org.cn/search/system/xgwj/2018/1205/194917.html |
| 32 | Regulations on the Work Concerning School Physical Education (2017 Version) | 2017 | SCPRC | http://www.gov.cn/gongbao/content/2017/content_5219126.htm |
| 33 | White Paper on the Development of China's Public Health as an Essential Element of Human Rights | 2017 | SCIO | http://www.scio.gov.cn/ztk/dtzt/36048/37159/index.html |
| 34 | The Plan for Promotion on Physical Education, Sports and Exercises of Children and Youth | 2017 | GASC, MOE, CCGCEP, NDRF, MCA, MOF, CYLC | http://www.sport.org.cn/search/system/gfxwj/qsnty/2018/1206/195012.html |
| 35 | Guiding Opinions on Accelerating and Advancing to Disseminate National Fitness Program into Families | 2017 | GASC, MCA, MOC, ACWF, CDPF | http://www.sport.org.cn/search/system/gfxwj/qzty/2018/1108/191882.html |
| 36 | Guiding Opinions on Strengthening the Work Concerning Physical Culture and Sports for Farmers | 2017 | MARA, GASC | http://www.sport.org.cn/search/system/gfxwj/qzty/2018/1108/191883.html |
| 37 | Guiding Opinions on Strengthening the Working Concerning Traditional Sports of Ethnic Minorities | 2018 | GASC, SEAC | http://www.sport.org.cn/search/system/gfxwj/qzty/2018/1108/191885.html |
| 38 | Physical Activity Guidelines for Preschool Children (3-6 years old) (Expert Consensus) | 2018 | BSU, CIP, CISS of GASC* | https://mp.weixin.qq.com/s/8kJZcGhSpFQ6jN55E56Jcg |
| 39 | Physical Activity Guidelines for Chinese Children and Youth | 2018 | SCMC, SUS, CHFU* | http://www.cnki.com.cn/Article/CJFDTotal-XZEK201706001.htm |
| 40 | Notice of Widely Promoting and Popularizing Radio Calisthenics | 2019 | GASC, SOWC of CPC, MOE, NHC, ACFTU, CYLC, CMG | http://www.sport.org.cn/search/system/gfxwj/qzty/2018/1204/308028.html |
| 41 | Guiding Opinions on Accelerating and Advancing National Youth Ice and Snow Sports into Schools | 2019 | MOE, NDRF, MOF, GASC | http://www.moe.gov.cn/srcsite/A17/moe_938/s3276/201906/t20190613_385735.html |
| 42 | Opinions on Deepening Education and Teaching Reform and Comprehensively Improving Quality of Compulsory Education | 2019 | CCCPC, SCPRC | http://www.gov.cn/zhengce/2019-07/08/content_5407361.htm |
| 43 | Opinions on Implementing the Healthy China Initiative | 2019 | SCPRC | http://www.gov.cn/zhengce/content/2019-07/15/content_5409492.htm |
| 44 | A Plan on the Implementation and Evaluation of the Healthy China Initiative | 2019 | SCPRC | http://www.gov.cn/zhengce/content/2019-07/15/content_5409499.htm |
| 45 | The Healthy China Initiative (2019-2030) | 2019 | HCPC | http://www.nhc.gov.cn/guihuaxxs/s3585u/201907/e9275fb95d5b4295be8308415d4cd1b2.shtml |
| 46 | Outline for Building a Leading Sports Nation | 2019 | GOSC | http://www.gov.cn/zhengce/content/2019-09/02/content_5426485.htm |
| 47 | Opinions on Promoting National Fitness and Sports Consumptions and Advancing Sport-industry’s High-quality Development | 2019 | GOSC | http://www.sport.org.cn/search/system/xgwj/2020/0221/310881.html |
| 48 | Guiding Opinions on Further Strengthening the Work Concerning Rehabilitation, Physical Health and Fitness for Persons with Disabilities | 2019 | CDPF, GASC | http://www.sport.org.cn/search/system/gfxwj/qzty/2020/0221/310892.html |
| 49 | Child and Youth Mental Wellness Action Plan (2019-2022) | 2019 | NHC, CCPPD, CCGCEP, OCCAC, MOE, MCA, MOF, NRTA, NWCCW, CYLC, ACWF, SOWC of CPC | http://www.nhc.gov.cn/jkj/tggg1/201912/6c810a8141374adfb3a16a6d919c0dd7.shtml |
| 50 | Law of the People's Republic of China on the Promotion of Basic Medical and Health Care | 2019 | NPCSC | http://www.npc.gov.cn/npc/c30834/201912/15b7b1cfda374666a2d4c43d1e15457c.shtml |
| 51 | Main Points of the Work Concerning Sports for All in 2020 | 2020 | GASC | http://www.sport.gov.cn/qts/n4986/c941449/content.html |
| 52 | Opinions on Deepening the Integration of Sports and Education to Promote the Healthy Development of Children and Youth | 2020 | GASC, MOE | http://www.sport.gov.cn/n316/n336/c963639/content.html |
| 53 | Opinions on Strengthening the Construction of National Fitness Facilities and Developing Sports for All | 2020 | GOSC | http://www.gov.cn/zhengce/content/2020-10/10/content_5550053.htm |
| 54 | Opinions on Comprehensively Strengthening and Improving the Work Concerning School Physical Education in the New Era | 2020 | GOCCCPC, GOSC | http://www.gov.cn/gongbao/content/2020/content_5554511.htm |
| 55 | Main Points of the Work Concerning Sports for All in 2021 | 2021 | GASC | https://www.sport.gov.cn/qts/n4986/c980367/content.html |
| 56 | Notice of the General Office of the Ministry of Education on further Strengthening the Physical Fitness and Health Management of Primary and Secondary School Students | 2021 | MOE | http://www.moe.gov.cn/srcsite/A17/moe_943/moe_947/202104/t20210425_528082.html |
| 57 | National Fitness Program (2021-2025) | 2021 | SCPRC | http://www.gov.cn/zhengce/content/2021-08/03/content_5629218.htm |
| 58 | The 14th Five-Year Plan for the Development of Physical Culture and Sports | 2021 | GASC | http://www.gov.cn/zhengce/zhengceku/2021-10/26/content_5644891.htm |
| 59 | Healthy Children Promotion Plan (2021-2025) | 2021 | NHC | http://www.gov.cn/zhengce/zhengceku/2021-11/05/content_5649019.htm |
| 60 | Physical Activity Guidelines for Chinese (2021) | 2021 | NHC, CCDC, CISS of GASC | http://chinaepi.icdc.cn/zhlxbx/ch/reader/create_pdf.aspx?file_no=20220102&flag=1&journal_id=zhlxbx&year_id=2022 |

**CCCPC:** The Central Committee of the Chinese Communist Party

**SCPRC:** The State Council of the People's Republic of China

**GASC:** General Administration of Sport of China

**MARA:** Ministry of Agriculture and Rural Affairs of the People's Republic of China

**MOE:** Ministry of Education of the People's Republic of China

**CYLC:** Central Committee of the Communist Youth League of China

**GOSC:** General Office of the State Council of the People's Republic of China

**ACFTU:** All-China Federation of Trade Unions

**MOC:** Ministry of Culture of the People's Republic of China

**NHC:** National Health Commission of the People's Republic of China

**CNCA:** China National Committee on Ageing

**NDRF:** National Development and Reform Commission of the People's Republic of China

**MOF:** Ministry of Finance of the People's Republic of China

**MCA:** Ministry of Civil Affairs of the People's Republic of China

**MLR:** Ministry of Land and Resources of the People's Republic of China

**MOHURD:** Ministry of Housing and Urban-Rural Development of the People's Republic of China

**PBOC:** People's Bank of China

**SAT:** State Administration of Taxation

**CBRC:** China Banking Regulatory Commission

**NTA:** National Tourism Administration

**ACWF:** All-China Women's Federation

**CDPF:** China Disabled Persons' Federation

**MIIT:** Ministry of Industry and Information Technology of the People's Republic of China

**MOHRSS:** Ministry of Human Resources and Social Security of the People's Republic of China

**MWR:** Ministry of Water Resources of the People's Republic of China

**GAC:** General Administration of Customs of the People's Republic of China

**SAIC:** State Administration for Industry and Commerce of the People's Republic of China

**NFGA:** National Forest and Grassland Administration

**CBIRC:** China Banking and Insurance Regulatory Commission

**NPCSC:** The Standing Committee of the National People's Congress

**SCIO:** The State Council Information Office of the People's Republic of China

**CCGCEP:** Central Commission for Guiding Cultural and Ethical Progress

**SEAC:** State Ethnic Affairs Commission of the People's Republic of China

**BSU**: Beijing Sport University

**CIP:** Capital Institute of Pediatrics

**CISS of GASC:** Sports Science Research Institute of the State Sports General Administration

**SCMC**: Shanghai Children's Medical Center

**SUS:** Shanghai University of Sport

**CHFU:** Children's Hospital of Fudan University

**SOWC of CPC:** State Organs Work Committee of Central Committee of the Communist Party of China

**CMG:** China Media Group

**HCPC:** The Healthy China Promotion Committee

**CCPPD:** The Publicity Department of the Central Committee of the Communist Party of China

**OCCAC:** Office of the Central Cyberspace Affairs Commission

**NRTA:** National Radio and Television Administration

**NWCCW:** National Working Committee on Children and Women under State Council

**GOCCCPC:** General Office of the Central Committee of the Chinese Communist Party

**NBDCP of NHC:** National Bureau of Disease Control and Prevention of National Health Commission of the People's Republic of China

**CCDC:** Chinese Center for Disease Control and Prevention
