# Supplementary material for: Analysis of national physical activity and sedentary behaviour policies in China
Source: BMC Public Health. 2023 May 30;23:1024. doi: 10.1186/s12889-023-15865-8 (PMC10230767; doi:10.1186/s12889-023-15865-8)
Supplement: Supplementary file 2 — Additional file 2: Number of Chinese physical activity and sedentary behaviour policies per issuing body/institution [file 12889_2023_15865_MOESM2_ESM.docx]

**
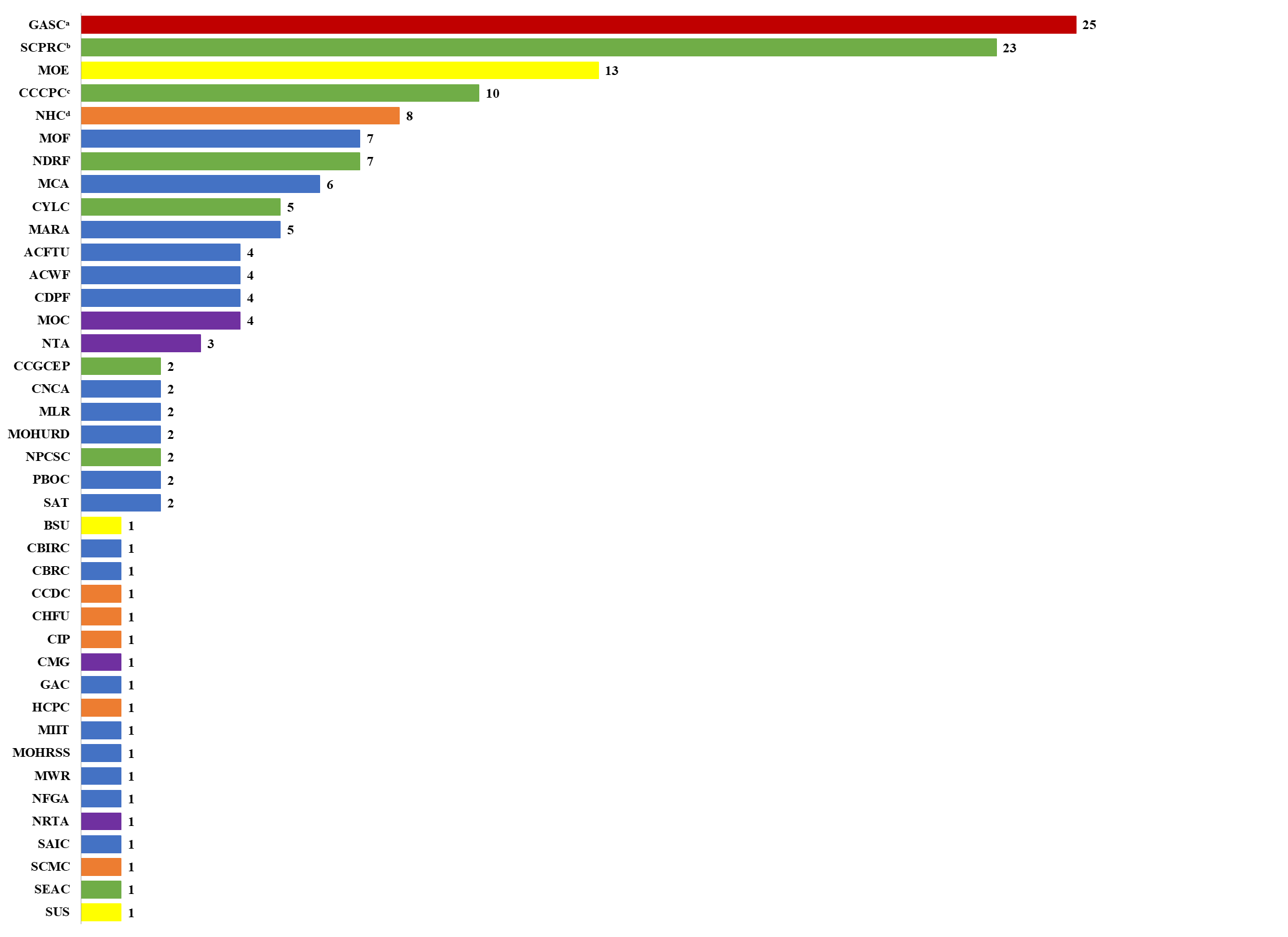
**

**Additional file 2. Number of Chinese physical activity and sedentary behaviour policies per issuing body/institution**

Green: central authority

Red: sports sector

Yellow: education sector

Orange: health sector

Purple: culture sector

Blue: other sectors

ACFTU: All-China Federation of Trade Unions

ACWF: All-China Women's Federation

BSU: Beijing Sport University

CBIRC: China Banking and Insurance Regulatory Commission

CBRC: China Banking Regulatory Commission

CCCCP^c^: The Central Committee of the Chinese Communist Party; ^c^ including CCPPD (The Central Committee of the Chinese Communist Party), GOCCCPC (General Office of the Central Committee of the Chinese Communist Party), OCCAC (Office of the Central Cyberspace Affairs Commission), and SOWC of CPC (State Organs Work Committee of Central Committee of the Communist Party of China)

CCDC: Chinese Center for Disease Control and Prevention

CCGCEP: Central Commission for Guiding Cultural and Ethical Progress

CDPF: China Disabled Persons' Federation

CHFU: Children's Hospital of Fudan University

CIP: Capital Institute of Pediatrics

CMG: China Media Group

CNCA: China National Committee on Ageing

CYLC: Central Committee of the Communist Youth League of China

GAC: General Administration of Customs of the People's Republic of China

GASC^a^: General Administration of Sport of China; ^a^ including CISS of GASC (Sports Science Research Institute of the State Sports General Administration)

HCPC: The Healthy China Promotion Committee

MARA: Ministry of Agriculture and Rural Affairs of the People's Republic of China

MCA: Ministry of Civil Affairs of the People's Republic of China

MIIT: Ministry of Industry and Information Technology of the People's Republic of China

MLR: Ministry of Land and Resources of the People's Republic of China

MOC: Ministry of Culture of the People's Republic of China

MOE: Ministry of Education of the People's Republic of China

MOF: Ministry of Finance of the People's Republic of China

MOHRSS: Ministry of Human Resources and Social Security of the People's Republic of China

MOHURD: Ministry of Housing and Urban-Rural Development of the People's Republic of China

MWR: Ministry of Water Resources of the People's Republic of China

NDRF: National Development and Reform Commission of the People's Republic of China

NFGA: National Forest and Grassland Administration

NHC^d^: National Health Commission of the People's Republic of China; ^d^ including NBDCP of NHC (National Bureau of Disease Control and Prevention of National Health Commission of the People's Republic of China)

NPCSC: The Standing Committee of the National People's Congress

NRTA: National Radio and Television Administration

NTA: National Tourism Administration

PBOC: People's Bank of China

SAIC: State Administration for Industry and Commerce of the People's Republic of China

SAT: State Administration of Taxation

SCMC: Shanghai Children's Medical Center

SCPRC^b^: The State Council of the People's Republic of China; ^b^ including GOSC (General Office of the State Council of the People's Republic of China), NWCCW (National Working Committee on Children and Women under State Council), and SCIO (The State Council Information Office of the People's Republic of China)

SEAC: State Ethnic Affairs Commission of the People's Republic of China

SUS: Shanghai University of Sport
