# Supplementary material for: Analysis of national physical activity and sedentary behaviour policies in China
Source: BMC Public Health. 2023 May 30;23:1024. doi: 10.1186/s12889-023-15865-8 (PMC10230767; doi:10.1186/s12889-023-15865-8)
Supplement: Supplementary file 3 — Additional file 3: Key elements for a successful national policy approach to physical activity promotion included in Chinese policies [file 12889_2023_15865_MOESM3_ESM.docx]

**Additional file 3. Key elements for a successful national policy approach to physical activity promotion included in Chinese policies**

| **Policy*** | **Element**^†^ | | | | | | | | | | | | | | | | | **Total**^‡^ |
| --- | --- | --- | --- | --- | --- | --- | --- | --- | --- | --- | --- | --- | --- | --- | --- | --- | --- | --- |
|  | 1 | 2 | 3 | 4 | 5 | 6 | 7 | 8 | 9 | 10 | 11 | 12 | 13 | 14 | 15 | 16 | 17 |  |
| **1** | ✓ | 🗶 | ✓ | 🗶 | 🗶 | 🗶 | ✓ | 🗶 | ✓ | ✓ | 🗶 | ✓ | ✓ | ✓ | 🗶 | 🗶 | 🗶 | 8 |
| **2** | ✓ | 🗶 | ✓ | 🗶 | 🗶 | 🗶 | ✓ | 🗶 | 🗶 | ✓ | ✓ | ✓ | ✓ | ✓ | 🗶 | 🗶 | ✓ | 9 |
| **3** | ✓ | ✓ | ✓ | 🗶 | 🗶 | 🗶 | ✓ | ✓ | 🗶 | ✓ | ✓ | ✓ | ✓ | ✓ | ✓ | ✓ | ✓ | 13 |
| **4** | ✓ | ✓ | ✓ | 🗶 | ✓ | ✓ | ✓ | 🗶 | 🗶 | ✓ | 🗶 | ✓ | ✓ | ✓ | ✓ | ✓ | 🗶 | 12 |
| **5** | ✓ | ✓ | ✓ | 🗶 | ✓ | 🗶 | ✓ | 🗶 | 🗶 | ✓ | ✓ | ✓ | ✓ | ✓ | ✓ | 🗶 | 🗶 | 11 |
| **6** | ✓ | 🗶 | ✓ | 🗶 | 🗶 | 🗶 | ✓ | 🗶 | 🗶 | ✓ | ✓ | ✓ | ✓ | ✓ | 🗶 | 🗶 | ✓ | 9 |
| **7** | ✓ | 🗶 | ✓ | 🗶 | ✓ | ✓ | ✓ | 🗶 | 🗶 | ✓ | ✓ | ✓ | ✓ | ✓ | 🗶 | 🗶 | 🗶 | 10 |
| **8** | ✓ | 🗶 | ✓ | 🗶 | 🗶 | 🗶 | ✓ | 🗶 | 🗶 | ✓ | ✓ | ✓ | ✓ | ✓ | ✓ | 🗶 | ✓ | 10 |
| **9** | ✓ | 🗶 | ✓ | 🗶 | 🗶 | 🗶 | ✓ | 🗶 | 🗶 | ✓ | ✓ | ✓ | ✓ | ✓ | 🗶 | 🗶 | ✓ | 9 |
| **10** | ✓ | ✓ | ✓ | ✓ | 🗶 | 🗶 | ✓ | 🗶 | 🗶 | 🗶 | 🗶 | 🗶 | ✓ | ✓ | ✓ | 🗶 | ✓ | 9 |
| **11** | ✓ | 🗶 | ✓ | 🗶 | ✓ | 🗶 | ✓ | ✓ | 🗶 | ✓ | 🗶 | ✓ | ✓ | ✓ | 🗶 | 🗶 | ✓ | 10 |
| **12** | ✓ | 🗶 | ✓ | 🗶 | 🗶 | 🗶 | ✓ | 🗶 | 🗶 | ✓ | 🗶 | ✓ | ✓ | ✓ | 🗶 | 🗶 | ✓ | 8 |
| **13** | ✓ | ✓ | ✓ | 🗶 | 🗶 | 🗶 | ✓ | ✓ | 🗶 | ✓ | ✓ | ✓ | ✓ | ✓ | ✓ | 🗶 | ✓ | 12 |
| **14** | ✓ | 🗶 | ✓ | 🗶 | 🗶 | ✓ | ✓ | 🗶 | 🗶 | ✓ | 🗶 | ✓ | ✓ | ✓ | ✓ | 🗶 | ✓ | 10 |
| **15** | ✓ | 🗶 | ✓ | 🗶 | 🗶 | 🗶 | ✓ | 🗶 | 🗶 | ✓ | 🗶 | ✓ | ✓ | ✓ | 🗶 | 🗶 | 🗶 | 7 |
| **16** | ✓ | 🗶 | ✓ | 🗶 | 🗶 | ✓ | ✓ | 🗶 | 🗶 | ✓ | ✓ | ✓ | ✓ | ✓ | 🗶 | 🗶 | ✓ | 10 |
| **17** | ✓ | ✓ | ✓ | ✓ | 🗶 | 🗶 | 🗶 | 🗶 | 🗶 | 🗶 | 🗶 | 🗶 | 🗶 | 🗶 | ✓ | 🗶 | ✓ | 6 |
| **18** | ✓ | ✓ | ✓ | 🗶 | ✓ | ✓ | ✓ | 🗶 | 🗶 | ✓ | ✓ | ✓ | ✓ | ✓ | 🗶 | 🗶 | ✓ | 12 |
| **19** | ✓ | 🗶 | ✓ | 🗶 | 🗶 | 🗶 | ✓ | 🗶 | 🗶 | ✓ | ✓ | ✓ | ✓ | ✓ | 🗶 | 🗶 | ✓ | 9 |
| **20** | ✓ | 🗶 | ✓ | 🗶 | 🗶 | 🗶 | ✓ | 🗶 | 🗶 | ✓ | ✓ | ✓ | ✓ | ✓ | ✓ | 🗶 | ✓ | 10 |
| **21** | ✓ | 🗶 | ✓ | 🗶 | 🗶 | 🗶 | ✓ | ✓ | ✓ | ✓ | ✓ | ✓ | ✓ | ✓ | 🗶 | ✓ | ✓ | 12 |
| **22** | ✓ | 🗶 | ✓ | 🗶 | ✓ | ✓ | ✓ | ✓ | ✓ | ✓ | ✓ | ✓ | ✓ | ✓ | 🗶 | 🗶 | ✓ | 13 |
| **23** | ✓ | 🗶 | ✓ | 🗶 | ✓ | ✓ | ✓ | 🗶 | ✓ | ✓ | ✓ | ✓ | ✓ | ✓ | 🗶 | 🗶 | ✓ | 11 |
| **24** | ✓ | 🗶 | ✓ | 🗶 | ✓ | ✓ | ✓ | ✓ | ✓ | ✓ | ✓ | ✓ | ✓ | ✓ | 🗶 | 🗶 | ✓ | 13 |
| **25** | ✓ | 🗶 | ✓ | 🗶 | ✓ | 🗶 | ✓ | 🗶 | 🗶 | ✓ | 🗶 | ✓ | ✓ | ✓ | 🗶 | 🗶 | ✓ | 9 |
| **26** | ✓ | ✓ | ✓ | 🗶 | ✓ | ✓ | ✓ | ✓ | ✓ | ✓ | ✓ | ✓ | ✓ | ✓ | 🗶 | ✓ | ✓ | 15 |
| **27** | ✓ | 🗶 | ✓ | 🗶 | 🗶 | ✓ | ✓ | 🗶 | 🗶 | ✓ | ✓ | ✓ | ✓ | ✓ | 🗶 | 🗶 | ✓ | 10 |
| **28** | ✓ | 🗶 | ✓ | 🗶 | 🗶 | 🗶 | ✓ | 🗶 | ✓ | ✓ | ✓ | ✓ | ✓ | ✓ | 🗶 | 🗶 | 🗶 | 9 |
| **29** | ✓ | 🗶 | ✓ | 🗶 | 🗶 | ✓ | ✓ | 🗶 | 🗶 | ✓ | ✓ | ✓ | ✓ | ✓ | 🗶 | 🗶 | ✓ | 10 |
| **30** | ✓ | 🗶 | ✓ | 🗶 | ✓ | ✓ | ✓ | 🗶 | ✓ | ✓ | ✓ | ✓ | ✓ | ✓ | 🗶 | 🗶 | ✓ | 12 |
| **31** | ✓ | 🗶 | ✓ | 🗶 | 🗶 | ✓ | ✓ | 🗶 | 🗶 | ✓ | ✓ | ✓ | ✓ | ✓ | 🗶 | 🗶 | ✓ | 10 |
| **32** | ✓ | 🗶 | ✓ | 🗶 | 🗶 | 🗶 | ✓ | 🗶 | 🗶 | ✓ | ✓ | ✓ | ✓ | ✓ | 🗶 | 🗶 | ✓ | 9 |
| **33** | ✓ | 🗶 | ✓ | 🗶 | ✓ | ✓ | 🗶 | 🗶 | 🗶 | ✓ | 🗶 | ✓ | ✓ | ✓ | 🗶 | 🗶 | ✓ | 9 |
| **34** | ✓ | 🗶 | ✓ | 🗶 | ✓ | ✓ | ✓ | ✓ | 🗶 | ✓ | ✓ | ✓ | ✓ | ✓ | 🗶 | 🗶 | ✓ | 12 |
| **35** | ✓ | 🗶 | ✓ | 🗶 | 🗶 | ✓ | ✓ | 🗶 | 🗶 | ✓ | ✓ | ✓ | ✓ | ✓ | ✓ | 🗶 | ✓ | 11 |
| **36** | ✓ | 🗶 | ✓ | 🗶 | 🗶 | ✓ | ✓ | 🗶 | 🗶 | ✓ | ✓ | ✓ | ✓ | ✓ | 🗶 | 🗶 | ✓ | 10 |
| **37** | ✓ | 🗶 | ✓ | 🗶 | 🗶 | 🗶 | ✓ | 🗶 | 🗶 | ✓ | ✓ | ✓ | ✓ | ✓ | 🗶 | 🗶 | ✓ | 9 |
| **38** | ✓ | ✓ | ✓ | ✓ | 🗶 | 🗶 | 🗶 | 🗶 | ✓ | 🗶 | 🗶 | 🗶 | ✓ | 🗶 | ✓ | 🗶 | ✓ | 8 |
| **39** | ✓ | ✓ | ✓ | ✓ | 🗶 | 🗶 | 🗶 | 🗶 | 🗶 | 🗶 | 🗶 | 🗶 | ✓ | 🗶 | ✓ | 🗶 | ✓ | 7 |
| **40** | ✓ | 🗶 | ✓ | 🗶 | 🗶 | 🗶 | ✓ | 🗶 | 🗶 | ✓ | ✓ | ✓ | ✓ | ✓ | 🗶 | 🗶 | ✓ | 9 |
| **41** | ✓ | 🗶 | ✓ | 🗶 | 🗶 | ✓ | ✓ | 🗶 | 🗶 | ✓ | ✓ | ✓ | ✓ | ✓ | ✓ | 🗶 | ✓ | 11 |
| **42** | ✓ | 🗶 | ✓ | 🗶 | 🗶 | 🗶 | ✓ | 🗶 | 🗶 | ✓ | ✓ | ✓ | ✓ | ✓ | 🗶 | 🗶 | ✓ | 9 |
| **43** | ✓ | ✓ | ✓ | 🗶 | ✓ | ✓ | ✓ | 🗶 | 🗶 | ✓ | ✓ | ✓ | ✓ | ✓ | 🗶 | 🗶 | ✓ | 12 |
| **44** | ✓ | 🗶 | ✓ | 🗶 | ✓ | ✓ | 🗶 | ✓ | 🗶 | ✓ | ✓ | ✓ | ✓ | ✓ | 🗶 | 🗶 | ✓ | 11 |
| **45** | ✓ | ✓ | ✓ | 🗶 | ✓ | ✓ | ✓ | ✓ | ✓ | ✓ | ✓ | ✓ | ✓ | ✓ | 🗶 | ✓ | ✓ | 15 |
| **46** | ✓ | 🗶 | ✓ | 🗶 | 🗶 | ✓ | ✓ | ✓ | ✓ | ✓ | ✓ | ✓ | ✓ | ✓ | ✓ | ✓ | ✓ | 14 |
| **47** | ✓ | 🗶 | ✓ | 🗶 | 🗶 | 🗶 | ✓ | ✓ | ✓ | ✓ | ✓ | ✓ | ✓ | ✓ | 🗶 | 🗶 | 🗶 | 10 |
| **48** | ✓ | 🗶 | ✓ | 🗶 | 🗶 | 🗶 | ✓ | ✓ | 🗶 | ✓ | ✓ | ✓ | ✓ | ✓ | 🗶 | 🗶 | ✓ | 10 |
| **49** | ✓ | 🗶 | ✓ | 🗶 | ✓ | ✓ | 🗶 | 🗶 | 🗶 | ✓ | ✓ | ✓ | ✓ | ✓ | 🗶 | 🗶 | ✓ | 10 |
| **50** | ✓ | 🗶 | 🗶 | 🗶 | 🗶 | 🗶 | 🗶 | 🗶 | 🗶 | ✓ | 🗶 | 🗶 | 🗶 | ✓ | 🗶 | 🗶 | 🗶 | 3 |
| **51** | ✓ | 🗶 | ✓ | 🗶 | 🗶 | ✓ | ✓ | ✓ | ✓ | ✓ | 🗶 | ✓ | ✓ | ✓ | 🗶 | 🗶 | ✓ | 11 |
| **52** | ✓ | 🗶 | ✓ | 🗶 | 🗶 | 🗶 | ✓ | ✓ | 🗶 | ✓ | 🗶 | ✓ | ✓ | ✓ | 🗶 | 🗶 | ✓ | 9 |
| **53** | ✓ | 🗶 | ✓ | 🗶 | 🗶 | 🗶 | 🗶 | 🗶 | 🗶 | ✓ | 🗶 | ✓ | ✓ | 🗶 | 🗶 | 🗶 | 🗶 | 5 |
| **54** | ✓ | 🗶 | ✓ | 🗶 | ✓ | ✓ | ✓ | ✓ | ✓ | ✓ | 🗶 | ✓ | ✓ | ✓ | 🗶 | 🗶 | ✓ | 11 |
| **55** | ✓ | 🗶 | ✓ | 🗶 | 🗶 | ✓ | ✓ | 🗶 | 🗶 | ✓ | 🗶 | ✓ | ✓ | ✓ | 🗶 | 🗶 | ✓ | 9 |
| **56** | ✓ | 🗶 | ✓ | 🗶 | ✓ | ✓ | ✓ | ✓ | ✓ | ✓ | 🗶 | ✓ | ✓ | ✓ | 🗶 | 🗶 | ✓ | 11 |
| **57** | ✓ | 🗶 | ✓ | 🗶 | 🗶 | ✓ | ✓ | ✓ | ✓ | ✓ | 🗶 | ✓ | ✓ | ✓ | 🗶 | 🗶 | ✓ | 11 |
| **58** | ✓ | 🗶 | ✓ | 🗶 | ✓ | ✓ | ✓ | ✓ | ✓ | ✓ | ✓ | ✓ | ✓ | ✓ | 🗶 | 🗶 | ✓ | 13 |
| **59** | ✓ | ✓ | ✓ | 🗶 | 🗶 | ✓ | ✓ | 🗶 | 🗶 | ✓ | ✓ | ✓ | ✓ | ✓ | 🗶 | 🗶 | ✓ | 11 |
| **60** | ✓ | ✓ | ✓ | ✓ | 🗶 | 🗶 | 🗶 | 🗶 | 🗶 | 🗶 | 🗶 | ✓ | ✓ | ✓ | ✓ | ✓ | ✓ | 10 |
| ***n***^§^ | 60 | 14 | 59 | 5 | 20 | 30 | 51 | 19 | 17 | 55 | 39 | 55 | 58 | 56 | 15 | 7 | 51 |  |

* The names of policies are provided in Additional file 1

^†^ Key elements for a successful national policy approach to physical activity promotion according to the health-enhancing physical activity policy audit tool (HEPA PAT – version 2):

1. Consultative approach in development

2. Evidence based

3. Integration across other sectors and policies

4. National recommendations on physical activity level

5. National goals and targets

6. Implementation plan with a specified time frame for implementation

7. Multiple strategies

8. Evaluation

9. Surveillance or health monitoring systems

10. Political commitment

11. Ongoing funding

12. Leadership and coordination

13. Working in partnership

14. Links between policy and practice

15. Communication strategy

16. Identity (branding/logo/slogan)

17. Network supporting professionals

^‡^ Number of elements in a given policy

^§^ Number of policies with a given element
